# Comparative cytogenetics of two endangered leuciscine fish, *Squalius aradensis* and *S. torgalensis* (Teleostei, Cyprinidae), from the Iberian Peninsula

**DOI:** 10.3897/CompCytogen.v7i1.4672

**Published:** 2013-03-18

**Authors:** Catarina Nabais, Massimiliano Rampin, Maria João Collares-Pereira

**Affiliations:** 1 Universidade de Lisboa, Faculdade de Ciências, Centro de Biologia Ambiental, Campo Grande, Lisboa, Portugal; 2 Present address: University of South Bohemia in České Budějovice, Faculty of Fisheries and Protection of Waters, Research Institute of Fish Culture and Hydrobiology, Vodňany, Czech Republic

**Keywords:** Leuciscinae, Cytotaxonomy, FISH with rDNA, NOR-phenotype, silver staining, chromomycin A_3_

## Abstract

In this study, the description of the karyotypes of the endangered chubs *Squalius aradensis* (Coelho, Bogutskaya, Rodrigues and Collares-Pereira, 1998) and *Squalius torgalensis* (Coelho, Bogutskaya, Rodrigues and Collares-Pereira, 1998) is presented by means of conventional (Giemsa-staining, Chromomycin A_3_ (CMA_3_)-fluorescence, Silver-impregnation (Ag-NORs)) and molecular (fluorescence *in situ* hybridization (FISH) with 18S rDNA probe) protocols. These endemic sister-species have an allopatric but adjacent distribution in the most southwestern part of the Iberian Peninsula. Diploid chromosome number was invariably 2n = 50 and karyotypes of both species were grossly similar, composed of metacentric and submetacentric elements with a reduced number of acrocentric pairs. Sequential staining using FISH with an 18S rDNA probe, CMA_3_ and Ag-NORs treatments revealed consistent positive signals located at the end of the short arms of a submetacentric chromosome pair, likely homologous in both species. While providing useful cytogenetic comparative data against other members of the genus *Squalius* Bonaparte, 1837, the work aimed to draw attention towards the conservation of two narrow-range and highly confined fish species.

## Introduction

The genus *Squalius* Bonaparte, 1837 belongs to the subfamily Leuciscinae, the major element of the Iberian cyprinid fauna. In Portuguese inland waters, four bisexual species with an allopatric distribution (Leunda et al. 2009) are recognized: *Squalius pyrenaicus* (Günther, 1868), in most drainages from the Tejo River basin southwards; *Squalius carolitertii* (Doadrio, 1988), from the most northern smaller drainages of the Atlantic slope to the Mondego River basin; *Squalius aradensis* (Coelho, Bogutskaya, Rodrigues and Collares-Pereira, 1998), in the Seixe, Aljezur, Alvor, Arade and Quarteira River basins; and *Squalius torgalensis* (Coelho, Bogutskaya, Rodrigues and Collares-Pereira, 1998), only present in the southern Mira River basin.

Most chubs of the genus *Squalius* were formerly included in the genus *Leuciscus* Cuvier, 1816 (see Sanjur et al. 2003). The two species with a wider geographic distribution, *Squalius carolitertii* and *Squalius pyrenaicus* (also present in Spain), were the first to be recognized as different taxonomic units. However, some genetic studies (Coelho et al. 1995, Brito et al. 1997) hypothesized the existence of a more complex differentiation pattern in the most south-western Iberian populations. Two new species, with a restricted geographic range, were then described by Coelho et al. (1998), *Squalius aradensis* and *Squalius torgalensis*,mainly based on meristic, morphometric and osteological traits. Further studies using in-depth mitochondrial and nuclear markers have confirmed that *Squalius aradensis* and *Squalius torgalensis* are separated, but sister-taxa (see Doadrio and Carmona 2003, Sanjur et al. 2003, Mesquita et al. 2005, 2007, Almada and Sousa-Santos 2010, Perea et al. 2010, Waap et al. 2011).

Some cytogenetic data were recorded for populations from Arade, Aljezur and Bordeira drainages (currently assigned to *Squalius aradensis*) and Mira (currently assigned to *Squalius torgalensis*) (Collares Pereira et al. 1998), but previously to the acknowledgement that they should be considered distinct taxa from *Squalius pyrenaicus* (Coelho et al. 1998). Besides, Collares-Pereira et al. (1998) reported higher karyotype variability in chubs belonging to southern populations, when compared to the northern drainages. Therefore, the present work aimed to describe the karyotypes of *Squalius aradensis* and *Squalius torgalensis* using specimens recently collected. Conventional (Giemsa staining, GC-specific CMA_3_ fluorescence and Ag impregnation) and molecular (rDNA major complex by FISH) cytogenetic techniques were used to characterize both species chromosome sets. Both endemics are “Critically Endangered” (Cabral et al. 2005) and besides an inherent heuristic nature, this work might draw attention towards the conservation of two narrow-range and highly confined fish species, while withdrawing useful cytogenetic comparative data against other members of the genus *Squalius*.

## Material and methods

Adult specimens were captured by electrofishing in two distinct southern populations. Six *Squalius aradensis* (four males and two females) were collected in the Arade basin (Odelouca River) and two *Squalius torgalensis* (unknown sex) in the Mira basin (Torgal River). All procedures were developed in accordance to the recommended ethic guidelines (ASAB 2006). Some old chromosome images from material collected in the same drainages and studied by Collares-Pereira et al. (1998) were also reanalyzed (2 specimens of *Squalius aradensis* and 3 specimens of *Squalius torgalensis*) in order to confirm the results obtained with the new material.

Chromosome spreads were obtained for one specimen using standard kidney protocol and for the remaining using fin fibroblast cultures (Rodrigues and Collares-Pereira 1996) to avoid fish sacrifice. Chromosome spreads were obtained by conventional splashing and selected for further cytogenetic analysis.

Chromosomes were stained with a solution of 4% Giemsa (pH=6.8). CMA_3_ fluorescence staining was performed according to Sola et al. (1992), with a slide pre-wash in McIlvaine/MgCl_2_ buffer, one hour incubation with CMA_3_ (Calbiochem) and Methyl green counterstaining. Ag-NORs detection followed Howell and Black (1980) with modiﬁcations (Gold and Ellison 1983), using Giemsa counterstaining. Whenever possible, slides were destained and used in sequential treatments.

The physical mapping of major rDNA gene cluster on the chromosomes was accomplished by FISH with rDNA probe. An rDNA clone containing 18S-5.8S-28S genes plus the intergenic spacers and untranscribed sequences from the genome of *Drosophila melanogaster* Meigen, 1830 (clone pDm 238, Rohia et al. 1981) was used as probe and labelled by nick translation with digoxigenin-11-dUTP according to the manufacturer’s specifications (Roche Applied Science). The probe was resuspended in hybridization mix composed of 50% ultra-pure formamide pH=7.5 (Sigma-Aldrich), 2× SSC and 10% (w/v) dextran sulfate powder (Sigma-Aldrich). Slides were denaturated in 70% formamide in 2× SSC at 65°C for 3 minutes, and immediately dehydrated in an ice-cold ethanol series (70%-90%-100%) for 7 minutes each and air-dried. The probe mixture was denatured at 75°C for 10 minutes, immediately placed on ice for another 10 minutes and added to the chromosome preparation. Hybridization was performed overnight in a dark moist chamber at 37°C. Post-hybridization washes were performed at room temperature (RT), for 7 minutes each: twice in 2× SSC and once in 2× SSC/0.1% Tween_20_. Slides were incubated with 3% bovine serum albumin (BSA) for 30 minutes at 37°C, in a dark moist chamber. Anti-digoxigenin antibody conjugated with fluorescein isothicyanate (FITC) (Roche Applied Science) was used to detect the probe for an hour and a half incubation at 37°C in a moist chamber. The slides were washed twice in 1x Phosphate Buffered Saline solution (PBS) at RT for 7 minutes and counterstained with DAPI in antifade solution.

Slides were screened in an Olympus BX 60 epifluorescence microscope equipped with a DP50 Olympus CCD camera. All images were processed using Adobe Photoshop CS4 software. Chromosomes were arranged in a decreasing size order and classified according to their arm ratios (Levan et al. 1964) in three categories: metacentric (m), submetacentric (sm) and sub-telocentric to acrocentric (st/a). To determine the fundamental number (NF value), chromosomes of the m and sm groups were considered biarmed and those of group st/a as uniarmed.

## Results

All *Squalius aradensis* and *Squalius torgalensis* karyotypes revealed a diploid number of 2n = 50 chromosomes.

**Figure 1.**
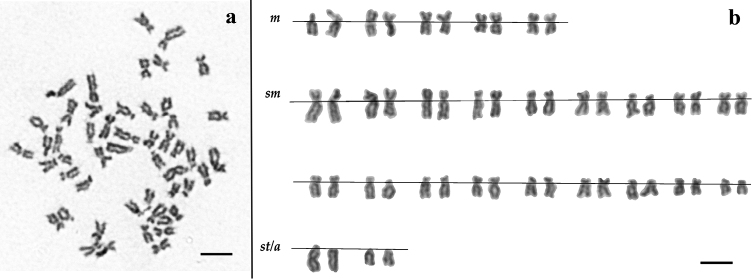
**a** Giemsa stained metaphase and **b** corresponding karyotype of a *Squalius aradensis* male from Odelouca River (Arade drainage). Scale bar = 10µm.

Karyotypes of both *Squalius aradensis* and *Squalius torgalensis* are composed of five pairs of metacentric (m), eighteen pairs of submetacentric (sm) and two pairs of subtelo/accrocentric (st/a) chromosomes. As the general karyotypes are the same for both species, only the results for *Squalius aradensis* using giemsa staining were included in Fig. 1. This genome composition leads to a high fundamental number (NF=96). As regards the eventual occurrence of heteromorphic sex chromosomes, no clear distinction between male and female karyotypes was observed in *Squalius aradensis*, the species where the specimens’ sex could be accurately assessed.

**Figure 2.**
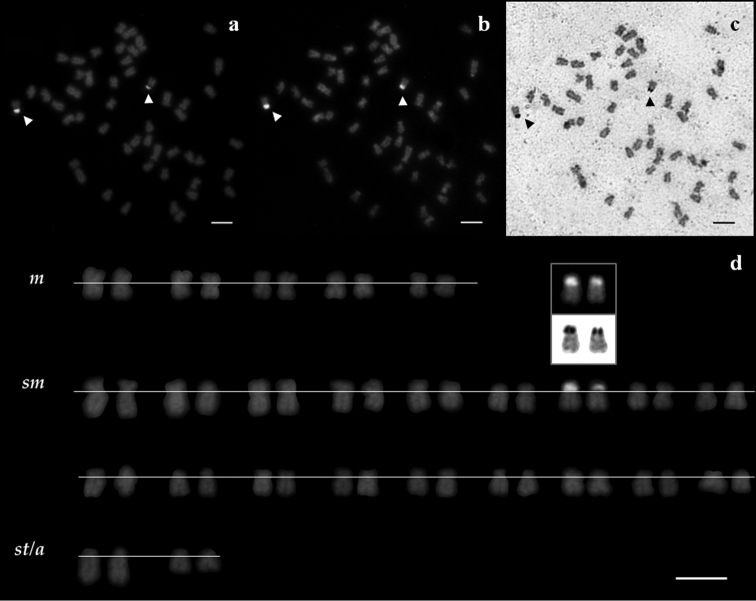
Metaphase spreads of a *Squalius aradensis* male, stained with sequential treatments **a** rDNA FISH **b** CMA_3_-NOR, and **c** Ag-NOR treatments **d** corresponding karyotype of the same individual after rDNA FISH, with CMA_3_- and Ag-NOR signals inset. NORs are indicated by arrowheads. Scale bar = 10µm.

**Figure 3.**
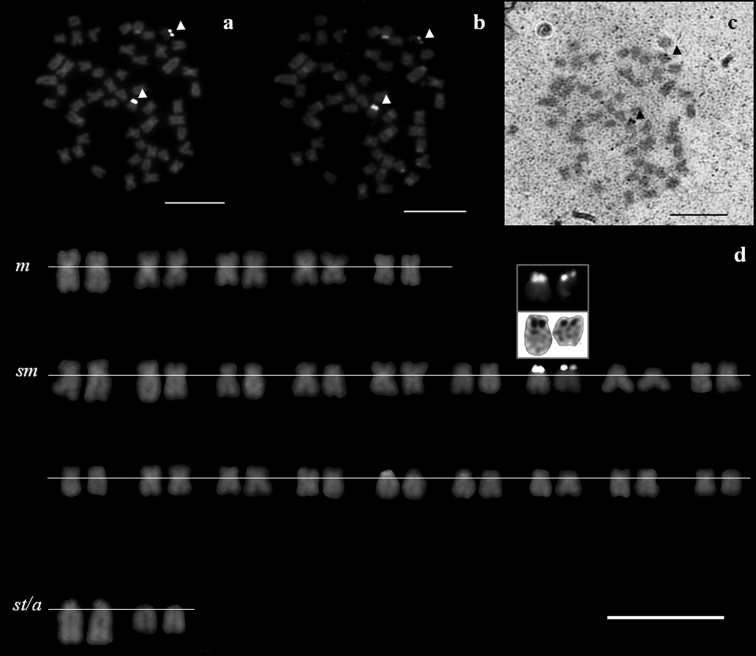
Metaphase spreads of a *Squalius torgalensis*, stained with sequential treatments **a** rDNA FISH **b** CMA_3_-NOR, and **c** Ag-NOR treatments **d** corresponding karyotype of the same individual after rDNA FISH, with CMA_3_- and Ag-NOR signals inset. NORs are indicated by arrowheads. Scale bar = 10µm.

The NORs’ phenotype was constant throughout all treatments, consistently positively labeling only one NORs-bearing chromosome pair likely homologous in both *Squalius aradensis* and *Squalius torgalensis* species. Their rDNA-positive signals were co-localized to CMA_3_- and Ag-positive signals: in the short arm of a middle-size sm chromosome pair in both species as documented by sequential staining (Figs 2 and 3), indicative of being GC-rich and transcriptional active. No evidences of multi-chromosomal positive NOR’s signals were registered.

## Discussion

*Squalius aradensis* and *Squalius torgalensis* karyotypes display the general pattern described for most Leuciscinae: a diploid chromosome number of 2n = 50, a chromosome set with mainly bi-armed elements (dominated by submetacentrics with some metacentrics), and only one NOR-bearing chromosome pair. In cyprinids this is assumed to correspond to the ancestral character state and most likely represents also the synapomorphy for the genus *Squalius* (Ráb and Collares-Pereira 1995). A large subtelocentric/acrocentric pair previously considered a chromosome marker for the subfamily (Ráb and Collares-Pereira 1995, Ráb et al. 2008) is also present in both species. The most common situation found in European Leuciscinae of a slightly higher number of st/a chromosomes might be due to the difficulty in obtaining high quality images for an accurate classification of chromosomes (Ráb and Collares-Pereira 1995, Collares-Pereira et al. 1998, Boron et al. 2009).

The number and location of rDNA gene clusters have been used as chromosome markers in fish cytotaxonomy (e.g. Rábová et al. 2003, Boron et al. 2009, Kirtiklis et al. 2010, Pereira et al. 2012, Rossi et al. 2012). Conversely to what was reported in *Squalius pyrenaicus* (Gromicho et al. 2005), no NORs polymorphism was observed in this study. Besides, Collares-Pereira et al. (1998) did not report any variation in NORs’ phenotype in all the populations analyzed. Karyotypes of these species conserved the plesiomorphic condition, where leuciscines have, in general, only one NOR-bearing chromosome pair (Ráb and Collares-Pereira 1995, Bianco et al. 2004, Luca et al. 2010, Rossi et al. 2012). Multiple NORs sites were detected in some *Squalius pyrenaicus* individuals using 28S rDNA FISH mapping, presenting intra-individual variation and also failure in Ag-NOR in detecting most rDNA copies (Gromicho et al. 2005). However, those observations were made in specimens living in sympatry with the *Squalius alburnoides* hybridogenetic complex (reviewed in Collares-Pereira and Coelho 2010) where hybridization is a recurrent process and might thus potentiate such variation in NORs.

The karyotypes of the two species proved to be grossly similar at a macrostructural level. They are sister-taxa strongly clustered in many phylogenetic analyses, however consistently reciprocally monophyletic (Almada and Sousa-Santos 2010, Waap et al. 2011). Given their differentiation estimated at 7-8 MY ago (Doadrio and Carmona 2003, Sousa-Santos et al. 2007, Almada and Sousa-Santos 2010), perhaps more subtle chromosome differences will be found when new cytogenetic tools with a higher resolution will be operating in cyprinid’s chromosomes. The clear segregation of their lineage to the one including *Squalius pyrenaicus* and *Squalius carolitertii* is also consensual in phylogenetic analyses, and tree topology strongly supports them as basal members of the south-western Iberian *Squalius* lineage. Taking that into consideration, it is not surprising the conservation of the plesiomorphic state of the karyotype in both *Squalius aradensis* and *Squalius torgalensis* but their differentiation to the other two species living in Portuguese inland waters, respectively with 10–12m + 30–32sm + 8st/a for *Squalius carolitertii*, and 12m + 32sm + 6st/a for *Squalius pyrenaicus* (Collares-Pereira et al. 1998).

Despite the apparent conservative pattern found in the two species here addressed by conventional cytogenetic tools, the karyotype variability present in the Iberian species of the genus *Squalius* so far analysed (Collares-Pereira et al. 1998, Gromicho et al. 2005), supports the occurrence of speciation processes favored by drastic changes in hydrological regimes (namely drought events), hence the difference between northern and southern populations. Whenever isolation and population bottlenecks occur, the sporadic mass reductions of population size might contribute to the stochastic fixation of chromosomal and genome mutations (Pereira et al. 2012). In particular, *Squalius torgalensis* is geographically confined to a single intermittent river system and characterized by a very low level of genetic diversity (see also Almada and Sousa-Santos 2010, Henriques et al. 2010). Thus specific conservation measures have to be adopted if the option will be to preserve the genome integrity of this highly vulnerable species.
